# Alternative stable ecological states observed after a biological invasion

**DOI:** 10.1038/s41598-022-24367-3

**Published:** 2022-12-02

**Authors:** Adriano G. Garcia, Walter Mesquita Filho, Carlos A. H. Flechtmann, Julie L. Lockwood, Juan A. Bonachela

**Affiliations:** 1 Department of Ecology, Evolution, and Natural Resources, Rutgers University, New Brunswick, 08901 USA; 2 Departamento de Entomologia e Acarologia, Escola Superior de Agricultura Luiz de Queiroz, Universidade de São Paulo (USP), Piracicaba, SP CEP 13418-900 Brazil; 3 Departamento de Fitossanidade, Engenharia Rural e Solos, Faculdade de Engenharia, Universidade Estadual Paulista (UNESP), Ilha Solteira, SP CEP 15385-00 Brazil

**Keywords:** Invasive species, Climate-change ecology, Theoretical ecology

## Abstract

Although biological invasions play an important role in ecosystem change worldwide, little is known about how invasions are influenced by local abiotic stressors. Broadly, abiotic stressors can cause large-scale community changes in an ecosystem that influence its resilience. The possibility for these stressors to increase as global changes intensify highlights the pressing need to understand and characterize the effects that abiotic drivers may have on the dynamics and composition of a community. Here, we analyzed 26 years of weekly abundance data using the theory of regime shifts to understand how the structure of a resident community of dung beetles (composed of dweller and tunneler functional groups) responds to climatic changes in the presence of the invasive tunneler *Digitonthophagus gazella*. Although the community showed an initial dominance by the invader that decreased over time, the theory of regime shifts reveals the possibility of an ecological transition driven by climate factors (summarized here in a climatic index that combines minimum temperature and relative humidity). Mid and low values of the driver led to the existence of two alternative stable states for the community structure (i.e. dominance of either dwellers or tunnelers for similar values of the climatic driver), whereas large values of the driver led to the single dominance by tunnelers. We also quantified the stability of these states against climatic changes (resilience), which provides insight on the conditions under which the success of an invasion and/or the recovery of the previous status quo for the ecosystem are expected. Our approach can help understand the role of climatic changes in community responses, and improve our capacity to deal with regime shifts caused by the introduction of exotic species in new ecosystems.

## Introduction

Ecologists have long had an interest in how and why abiotic or biotic drivers can lead to changes in the resilience of a specific ecosystem state, and under what circumstances such drivers will result in an ecosystem potentially moving into a different stable state^2–4^. There is evidence that such ecological regime shifts can be driven by climatic factors like increased warming, heat waves, and droughts^5,6^. In addition, climate can interact with co-occurring anthropogenic disturbances such as invasive species^7–9^, increasing the per-capita effect of the invader on native species in some situations^10^, which may directly or indirectly shift the ecosystem to a new state^11^. The understanding of how invasive species affect changes of dominance triggered by climate factors is, however, still lacking. In this context, investigating a particular example can reveal important information on the climatic resilience of an ecosystem to the modifications brought by an invasive species. To address this knowledge gap, we used the theory of regime shifts to explore the climatic response of a dung beetle community in Brazil after the invasion of a competitor^1^. Specifically, our goal was to understand the response of the dung beetle community to climate in the presence of the strongly interacting invader, including the characterization of community structure, the possibility for alternative stable states, and their resilience.

Although the theory behind resilience and regime shifts has matured considerably in recent years^12^, empirical evidence of ecological transitions has continued to lag, leading to a debate on their prevalence and importance^13,14^. The current ‘gold standard’ to provide empirical evidence for regime shifts comes from a combination of empirical observation and experimentation or modeling^8,15^. The increasing availability of long-term datasets has provided another compelling avenue of investigation, especially in regards to how ecosystems respond to combinations of documented changes in system drivers^5,9,16^, although a challenge in the identification of an ecosystem’s stable states and shifts between them is still the absence of field studies, since laboratory observations are not always verified in non-controlled situations^17^.

Here, we analyzed a previously published 26-year dataset on a focal native dung-beetle community after the introduction of the non-native *Digitonthophagus gazella* (Fabricius, 1787). *D. gazella* was (allegedly) intentionally introduced in Brazil aiming to reduce the negative effects of cow pads within grazed lands and control dung-breeding horn flies^18,19^. Dung beetles provide substantial ecosystem services within open-grass dominated ecosystems^20,21^. For example, their decomposition activity leads to a reduction of natural greenhouse gas emissions (especially methane) by up to 40%^22,23^; moreover the burial of manure deposited in grazing systems is followed by nutrient cycling, increasing grass growth and fertilization^22,24^.

The functional composition of a dung beetle community is key to the provisioning of these services^25^, and therefore shifts in this functional diversity are of clear practical importance in Brazil and elsewhere. Thus, here we used functional diversity to represent the state of our focal dung beetle community. The community was composed of two functional groups: dwellers, which do not move the dung from its original place but consume the food directly in the dung piles; and tunnelers, which excavate tunnels and store the food underground^26,27^. The presence of *D. gazella* had a clear effect on functional redundancy within our focal system, as it replaced nearly all native species of its same functional group (tunnelers) and suppressed the abundance of the other (dwellers, see below). During the same time frame, local climatic conditions shifted toward drier and warmer conditions in our focal region, likely in accordance with the El Niño Southern Oscillation^1^. Each dung beetle group in this community exhibits a certain response to these climatic factors (see below), and therefore it is expected that the diversity of the dung beetle community also shift in accordance with prevailing climatic conditions. Mesquita Filho et al.^1^ indeed found that several climatic factors drive each group’s changes over time. Here, using the tools and concepts from the theory of regime shifts, we characterized the qualitative and quantitative response of the community to climatic changes by defining a single variable representing community structure (system state), and a single index representing climate. Although intermediate values of the climatic index should facilitate the survival of both groups, it is unclear whether this will translate into a single state in which one group dominates over the other, or the system can alternate dominance by either group. It is also unclear whether there is a climate range that favors overwhelming dominance by one group, and what that range is. To fill this gap, we explored whether the focal system transitioned between states, and measured the resilience of the associated states. Our framework contributes to understanding the environmental conditions that, in the presence of an invasive species, may trigger long-term changes in the community. In addition, our methodology may provide important information to support management plans, thus increasing the capacity to deal with these regime shifts and reduce the likelihood of disruptive events^7,28–30^.

## Methods

### Study system

Our focal ecosystem is in Selvíria, state of Mato Grosso do Sul, Brazil ($\hbox {20}^{\circ }$ $22'$ $41.86''$ S, $\hbox {51}^{\circ }$ $24'$ $58.90''$ W), on a property owned by the São Paulo State University (UNESP). The location covers 350 ha of pasture composed of liverseed grass (*Urochloa decumbens*). The native vegetation was removed, pasture areas were implemented, and ﻿livestock was introduced in the 1970s, maintaining this configuration during the following 50 years. The climate of this area is categorized as equatorial savanna, with dry periods concentrated mostly during the winter, from April to August. During our sampling period (from November 23th, 1989, to November 19th, 2015), no vermifuges and insecticides that could affect negatively the community of dung beetles associated with cow pads were used^1^.

**Figure 1:**
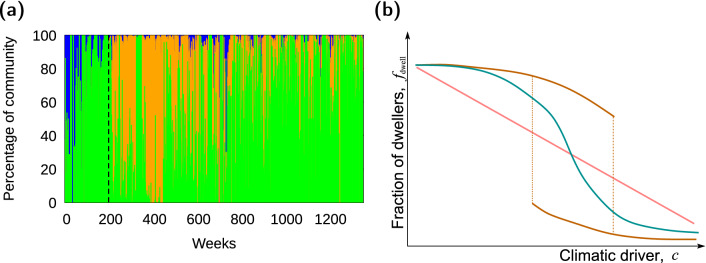
Left: Community composition by functional group for all weeks of observation^1^. Green represents dwellers, blue represents tunnelers, and orange represents the invader *D. gazella*. Right: Sketch of responses of the community composition to the climatic driver (i.e. phase diagram) expected from the physiological and behavioral characteristics of the functional groups in the community as described in text: linear (red), or non-linear but monotonic without (blue) or with (brown) hysteresis.

The native dung beetle community at this site is composed of dwellers and tunnelers. Dwellers comprise the Aphodiinae subfamily, whereas all the tunnelers belong to the Scarabaeinae subfamily^31^. In total, there were eight species classified as dwellers (*Ataenius crenulatus*, *A. picinus* and *Atanius aequalis-platensis* grouped as one species, *Blackburneus furcatus*, *Genieridium bidens*, *Labarrus pseudolividus*, *Nialaphodius nigrita* and *Trichillum externepunctatum*) and ten native tunnelers (*Ateuchus* nr. *puncticollis, A. vividus*, *Canthidium nr. pinotoides*, *Dichotomius bos*,* D. semiaeneus*, *D. sexdentatus*, *Ontherus appendiculatus*, *O. dentatus*, *O. sulcator*). These species were chosen for our study because, as the invasive tunneler *D. gazella* (also from the Scarabaeinae subfamily), they all co-occur in pasture and exploit the same resource (cow pad)^32^. The initial establishment of *D. gazella* caused the loss of most of the native tunnelers from the community, with the invader becoming the overwhelming representative of the functional group, and an initial decrease of abundance for dwellers. Differently from native tunnelers, however, dwellers were able to recover their number a few years after invasion (Fig. 1a, Fig. S1).

As reported in^1^, the abundance of dung beetles was significantly affected by both local minimum temperature and relative humidity. The influence of these two factors is expected, as they determine egg and larval survival and development of dung beetles. For example, because dung beetles are poikilotherms, environmental temperature is key to their development and fecundity^33^. One of the main dweller species, *Labarrus pseudolividus*, is widely found in locations with temperature averages ranging between $\hbox {12}\,^{\circ }\hbox {C}$ and $\hbox {18}\,^{\circ }\hbox {C}$^34^, making it tolerant to colder local temperatures. On the other hand, for *D. gazella* the lower developmental threshold is $\hbox {15.5}\,^{\circ }\hbox {C}$ (individuals cannot survive below this temperature), and the optimum temperature for population growth is $\hbox {28}\,^{\circ }\hbox {C}$^35^. For both groups, physiological growth and reproduction rates are maintained even when outside temperatures are close to the lower developmental threshold; dwellers, for example, live inside the dung pile, where temperature is higher and less variable than outside^36,37^. However, while tunnelers oviposit deep in the soil to protect the eggs, warmer and drier conditions reduce dweller egg viability on dung piles since they are exposed^38^. Low humidity conditions lead to drier dung and can cause egg and insect dessication. In addition, dwellers from our focal system have Palearctic evolutionary origins^39^; *D. gazella*’s natural distribution ranges from central to southern Africa^40^, presenting high physiological plasticity that allows it to tolerate high temperatures and low relative humidity better than other tunneler species^41^.

### Functional-group data collection and community structure characterization

Dung beetles were collected once a week in a black-light flight intercept trap^42^, which guarantees the collection of coprophagic beetles. During all collection periods, climate variables were also collected from a meteorological station located within 2 km of our collecting site. See^1^ for the complete description of the collection process and database. For our purposes, we retained the species, number of individuals per species, and climate variables for each week sampled (Supplementary Information, SI, Figs. S1–S2).

We focused first on the weekly abundance data, which we needed to process in order to avoid spurious results in our analyses stemming from the measurement protocol. Specifically, we filtered out seasonal low values associated with sampling in the coldest periods, when few beetles are captured because the reduced activity in all functional groups restricts their spatio-temporal distribution^43^. Including such samples would not be representative of the community and could bias the analysis since we are investigating community composition (i.e. proportions, very sensitive to low sampling). Thus, we considered only samples with a total number of beetles (that is, summing up all groups together) higher than the value of the median of all data, a conservative threshold that retains observations that allow for as much representation of the community as possible. As will become evident in the Results section and Supplementary Information, less conservative choices for the threshold did not alter our main conclusions.

Following Mesquita -Filho et al.^1^, we categorized all sampled species into either dwellers or tunnelers. *D. gazella* is a tunneler and, as explained above, the native tunneler species experienced massive declines in abundance after its establishment, leaving *D. gazella* as almost the single representative in the tunneler functional group during the period of observation^1^. Thus, given the sharp contrast in community composition, we also separated the data into before and after invasion using to that end the 200th week, when *D. gazella* was first observed at the study site (September 11th, 1993, starting date for what we will call “after invasion”, our focal period henceforth).

To describe community functional composition (i.e. system state) through time, we derived a normalized functional group ratio. First, because the abundance of each functional group spanned up to four orders of magnitude, we performed a logarithmic transformation of the number of captured insects from each group *i*, $\log _{10}(N_{i}+K)$, following  Yamamura^44^. Here, we chose $K=1$, but the value of *K* did not alter our results qualitatively. In addition, the original data showed random mismatches in the phenology of each group, which gave the wrong impression of extreme short-term shifts in functional group dominance within the community. To avoid such artifacts, we used nonparametric local regression (LOESS)^45^ to smooth the dynamics of each group^46^. For this smoothing, we employed the *loess* function in the R software 3.6.1^47^ with a smooth parameter equal to 0.25, but other moderate values (or an optimal value calculated with Bayesian inference by the R function *optimal_span*) did not alter our conclusions. Finally, we extracted back from the smoothed curve the number of beetles within each functional group to calculate the fraction $f_{dwell}$ that measures the relative abundance of dwellers:

1 $$\begin{aligned} f_{dwell} = \frac{N_D}{N_D+N_T} \end{aligned}$$

where $N_D$ corresponds to the number of dwellers per week and $N_T$ corresponds to the number of native tunnelers (for the period before invasion), or only the number of *D. gazella* observed per week (after invasion), using their corresponding smoothed curves. Including also native tunnelers after invasion did not alter our conclusions.

### Climate driver

We devised a single climatic driver variable that merges the weekly measurement of temperature and relative humidity over the years, abiotic factors key to the survival and reproduction of both groups (see above). We first converted minimum temperatures and relative humidity to normalized climate variables using a min-max normalization (a feature scaling that uses the total range of temperatures or relative humidity, respectively, as normalization factor):

2 $$\begin{aligned} T = \frac{T_{week} - T_{min}}{T_{max}-T_{min}}\;\;,~ ~ ~ ~ ~ ~ RH = \frac{RH_{week} - RH_{min}}{RH_{max}-RH_{min}}\;\;, \end{aligned}$$

where *T* corresponds to the normalized temperature, $T_{week}$ is the weekly temperature, and $T_{max}$ and $T_{min}$ are the absolute maximum and minimum temperatures observed during the whole sampling period, respectively. We used a similar notation for relative humidity, *RH*. Based on the information above regarding beetle response to climate, the merged climate factor *c* was defined as the relationship:

3 $$\begin{aligned} c = \frac{T}{RH}\;\;, \end{aligned}$$

for $RH\ne 0$. That is, higher temperatures and/or drier conditions (expected to favor *D. gazella*) lead to higher values for *c*. On the other hand, lower temperatures and/or more humid conditions (expected to favor dwellers) imply lower values for *c*. Intermediate values of *c* can represent either moderate or extreme values for *both* *T* and *RH*.

### Identifying ecological states and quantifying resilience

With our $f_{dwell}$ data as an index of community composition (i.e. system state), we calculated kernel density functions to interpolate a continuous probability distribution of the relative fraction of dwellers in the community, $p_{n}(f_{dwell})$ (function *density*, R software 3.1.6^47^) for a given range of climatic driver *c* values. We grouped the $f_{dwell}$ data using ranges for *c* of size 0.4, to ensure a significant amount of weekly samples that allowed for the reconstruction of these probability distributions (see Table S1, first column). Note that bins with extreme values showed few data points (see first and last rows in Table S1), and thus were rejected to prevent misleading results due to reduced sampling. Also note that, for the *density* function, we used the default Gaussian kernel with a smoothing bandwidth adjusted to be $50\%$ larger than the default value (“adjust” argument set to 1.5). This conservative choice aims to reduce the effect of the different sampling across *c* bins and to ensure that differences among distributions across *c* values are not the result of spurious sampling noise.

Further, we transformed the kernel density function:

4 $$\begin{aligned} V(f_{dwell}) = -\ln (p_{n}(f_{dwell})) \end{aligned}$$

This $V(f_{dwell})$ function, called potential (e.g.^48^), shows by design well-defined minima for the most frequently observed values of $f_{dwell}$ (i.e. configurations most frequently observed for the community, which conform the modes of the probability distribution) in a given group of data. At these points, the potential exhibits a change of trend from decreasing to increasing, and therefore its derivative shows a change of sign. Eq. (4), thus, provides a simple criterion to identify possible system states, which is a reason why potentials have been used extensively across disciplines^49–51^. Nonetheless, because the position of extrema is invariant under the transformation, using probability distributions instead would not alter our conclusions.

Representing the potential obtained from all the $f_{dwell}$ system states associated with a same range of climatic driver *c* values allowed us to identify stable community configurations associated with a specific climate. The comparison of the potentials obtained for different *c* ranges enabled the description of how the community changed in response to climatic variation. The location of the minima revealed which states were stable for a given value of the climatic driver; the presence of two minima, then, flagged the existence of bistability (i.e. two different community compositions possible for the same *c* value).

These minima are materialized as wells in the potential’s landscape, which provides an easy way to understand the concept of stability: the dynamics of the system for the given value of the driver will eventually “fall” into a well (either a state dominated by dwellers or a state dominated by tunnelers), with the shape of the well (e.g. its depth) determining how difficult it is for the system to “escape” that state. Therefore, the area inside a well provides quantification of the tendency of a system to stay in that specific state, i.e. the resilience of the associated ecological state or how strong a perturbation has to be to move the system from such an ecological state to another^2,3,50–53^. Thus, in addition to number and location of wells, measuring their associated area allowed us to further characterize the resilience of the community. To this end, we first set a visualization window common to all potentials. Specifically, we plotted the potentials within a range for the vertical variable (the potential, *V*) given by $[-1.5,1.5]$; the horizontal variable (fraction of dwellers, $f_{dwell}$) is by definition bounded between 0 and 1. For potentials that showed one single well, the area of the well was measured as the area above the potential curve within this visualization window. For potentials that showed two wells (bistability), we measured the value of the potential at the local maximum separating the two wells, and established that value as the upper (horizontal) line closing the area of each well. To ensure all cases were comparable and eliminate any arbitrariness of the choices above, we expressed resilience as a relative area; in other words, we further normalized the well area by the total area across wells for that potential, which means that any single-well case will show a resilience (or relative area) of 1, and the resilience of the two wells when there is bistability adds up to 1.

### Identifying ecological transitions

Measuring a state variable, $f_{dwell}$, and a driver, *c* (order and control parameter, respectively, in the jargon of regime shift theory), allowed us to study how their observed behavior over time materializes in a driver-state relationship (the so-called phase diagram) defining the possible shifts in dominance (i.e. regime shifts) that the community may undergo as climate changes^12^. The non-monotonic temporal behavior of the components of the order parameter (i.e. dwellers and tunneler availability) and the components of the control parameter (i.e. temperature and relative humidity) makes it difficult to predict the shape of the phase diagram, and therefore whether we can expect alternative stable states in the focal example. For such cases, the dominance of the dung beetle community could (1) shift in a linear fashion toward the functional group favored by climatic conditions; (2) shift between functional groups in non-linear threshold response to climatic conditions without hysteresis; or (3) shift between functional groups in non-linear threshold response to climatic conditions with hysteresis –and thus showing bistability (see Fig. 1b, or^12^). Other possibilities, e.g. a non-linear shift between functional groups where one group is favored at intermediate climatic conditions^12^ are discarded as the invader is better suited for warmer and drier conditions. To evaluate which of these possibilities occurred, we represented $f_{dwell}$ as a function of *c*, as well as the location of the minima shown by the potentials above. In addition to the emerging shape of this relationship, this plot can reveal the presence of alternative stable states if two or more different points occur for the same value of the control parameter, *c*.

## Results

We calculated the median of all weekly sample sizes ($N_{median}=59$ individuals, see Fig. S1), a value that was used as the threshold to filter our data as described above. This value was appropriate considering the range of the data, because it preserved the information from months when beetles are more active and thus when sampling provides a reliable snapshot of the community.

### Characterization of the state of the system and climatic driver

**Figure 2:**
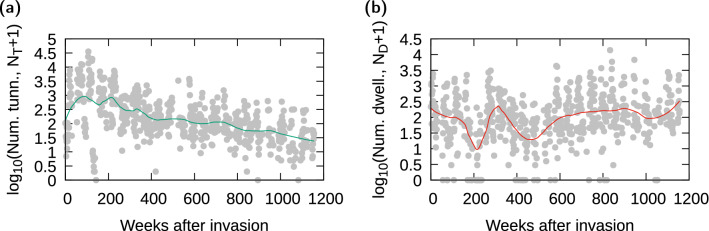
Log-transformed number of individuals for both native and invasive tunneler *D. gazella* (left) and dwellers (right) as a function of time after the invasion. The curve through the points was obtained using a nonparametric local regression smoother (loess) with smoothing intensity 0.25.

The abundance of *D. gazella* quickly increased after being introduced then started to slowly decrease after $\sim 200$ weeks (Fig. 2a). Over the same time frame, dwellers initially declined then recovered slowly but in an irregular fashion after the invader’s peak (Fig. 2b).

**Figure 3:**
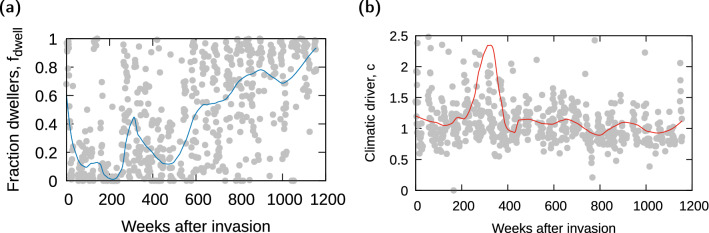
(**a**) Change with time (weeks after invasion) of the fraction of dwellers, $f_{dwell}$ (blue line representing the loess version); note that the order parameter is calculated already from the smoothed data (see Fig. 2 and main text), but overimposing also the fraction of dwellers calculated before smoothing $N_{D}$ and $N_{T}$ (gray dots) provides a sense for the degree of variation observed. (**b**) Change with time of climate observable, *c* (gray dots), with its corresponding loess line (red).

The fraction of dwellers showed a sharp decline after *D. gazella* was introduced at the study site (Fig. 3a). It reached a minimum around week $\sim 200$ after invasion, increasing and decreasing in the following $\sim 300$ weeks, and increasing monotonically afterwards. Thus, $f_{dwell}$ summarized accurately the behavior of the community inferred from the functional group abundance trends.

Our climate driver variable, *c*, summarized the temperature and relative humidity data at the study site (Fig. S2 and Fig. 3b). After invasion, *c* presented a slight overall downward trend. The overall range of variation was similar before and after invasion (Fig. S3), although the (much longer) period after invasion showed more marked fluctuations and outlier values.

### Characterization of the stability landscape (potential)

**Figure 4:**
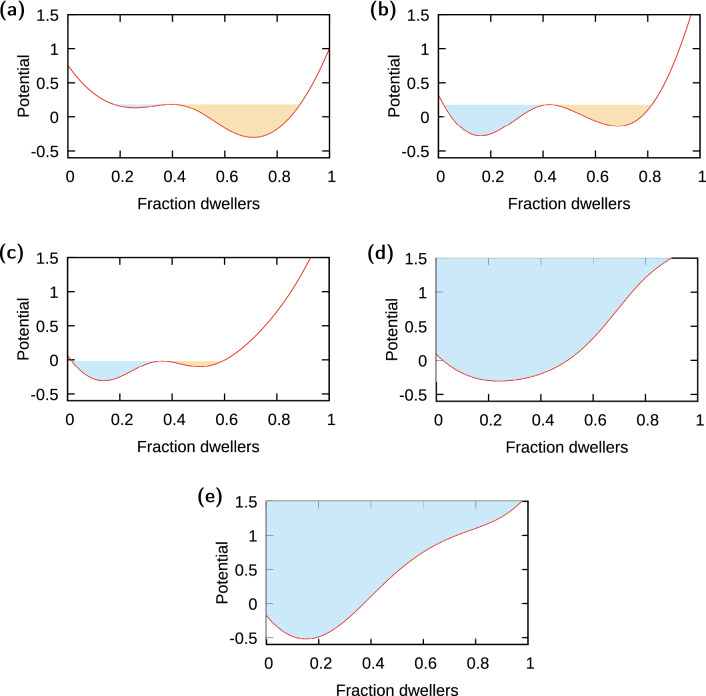
Potentials for different values of *c*, calculated as explained in the main text. Note that, unless two *true* minima (i.e. locations on the curve where the derivative is zero) are detected, the area is calculated as the total area shown by the single well. (**a**) Climatic driver $c=[0.4, 0.8 ]$; (**b**) Climatic driver $c=[0.8, 1.2 ]$; (**c**) Climatic driver $c=[1.2, 1.6 ]$; (**d**) Climatic driver $c=[1.6, 2.0 ]$; (**e**) Climatic driver $c=[2.0, 2.4 ]$. See Table S1 for the corresponding ranges of temperature and relative humidity.

We then classified the $f_{dwell}$ data according to their associated *c* value, and calculated the associated potential (Fig. 4). The shape, location, and number of minima of the potential changed with the climatic driver *c*. A well around a value $f_{dwell}>0.5$ indicated a dweller-dominated state, whereas a well around a value $f_{dwell}<0.5$ indicated a invader-dominated state. In the period after invasion, we observed a single well for large *c*, characterized by a decreasing $f_{dwell}$ value ranging between 0.10 and 0.30; we observed two minima for any other *c* (one well around $f_{dwell} = 0.17{-}0.19$ and another well around $f_{dwell} = 0.66{-}76$, or $\sim \,0.5$ if *c* within $[1.2,\, 1.6]$).

**Figure 5:**
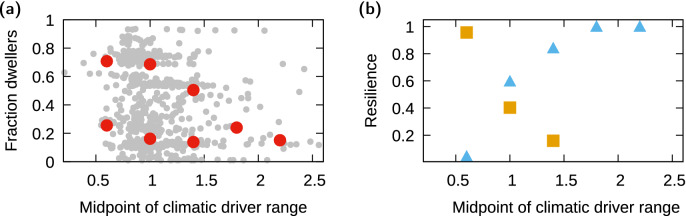
(**a**) Phase diagram, i.e. relationship between the order parameter, $f_{dwell}$, and the climatic driver, *c*, after invasion; red points represent the location of the minima in the associated potentials (see Fig. 4) and thus two dots for the same *c* range indicate two alternative stable states, i.e. bistability of both functional groups. (**b**) Normalized area of the wells in the different potentials; squares represent the dweller-dominated well and triangles the tunneler-dominated well.

Representing the values of the minima against the value of the driver confirms that, after *D. gazella* was introduced, the invasive tunneler dominated the community for high *c*, and the system showed dominance by either group for intermediate and low values of the climatic driver (Fig. 5). As Fig. S8 shows, the existence of bistability was robust against different choices for the smoothing parameter and against different choices for the threshold below which we consider a sample not representative of the community (see above).

Finally, we measured the area of each well shown by the potentials (shaded area in Fig. 4), and normalized by the total area across wells. We then represented this measure of stability as a function of the climatic driver. After invasion, the area of the well representing the dweller-dominated state decreased with *c* while that of the invader-dominated state increased (Figs. 4 and 5b).

### Behavior before invasion

Before invasion, the abundance of dwellers increased whereas that of tunnelers decreased (Fig. S4). The fraction of dwellers increased over time (Fig. S5a), although no trend is discernible for the climatic driver before the introduction of *D. gazella* (Fig. S5). The pre-invasion community showed only one well for the associated potential (Fig. S6), and the location of the single well was at a $f_{dwell}$ value that increased with *c* but always remaining above 0.80, thus indicating clear dominance by dwellers (Fig. S7) for any value of the climatic driver *c*. The area of the single well remained barely changed across values of the climatic driver (Fig. S7). The period before invasion showed, because of the scarcity of data available, much more sensitivity to both smoothing and threshold parameters.

## Discussion

Identifying and characterizing ecological transitions, and the possibility of alternative stable states associated with the transition, is an elusive but essential task. Given the accelerated rate of climatic change, understanding how potential climatic drivers affect a focal system, and whether they trigger a significant change in its observed state (i.e. a transition or shift) or lead to bistability, is key to understanding system dynamics and informing management decisions. Identifying the control and order parameters of the transition provides invaluable information to this end, but requires a large amount of data. Here, the use of a 26-year data collection in combination with the theory of regime shifts has unveiled a climate-driven dominance transition and alternative stable states in a beetle community in the presence of the invasive *D. gazella*. Although the temperature and relative humidity before and after invasion were within similar ranges, an extended period of time with drier conditions occurring after the establishment of *D. gazella* and higher temperatures (leading to high *c* values) favored the invasive species (a tunneler), allowing it to effectively exclude most native tunnelers and dominate the community. Intermediate climatic ranges led to the bistability of alternative states where either dwellers or tunnelers dominated. This reveals the existence of an abrupt transition (or shift in dominance) triggered by the combined effect of temperature and relative humidity.

### Changes in dominance driven by climate

We identified an ecologically relevant parameter able to represent the state of the community ($f_{dwell}$, order parameter), and a parameter able to encapsulate the main environmental drivers of the transition (*c*, control parameter). These two measures provided a clear picture of the qualitative changes in beetle functional composition after invasion under different climate conditions. After invasion, the dominance of tunnelers or dwellers strongly depended on *c*.

Our results illustrate how the establishment of the invader, involving survival and reproduction, required for the organism to overcome a “survival barrier”^54^. Our framework allowed for the identification of such a barrier, which in our case is values of the composite driver $c>0.4$. With low presence for weeks after introduction, the invasive tunneler reached dominance only after the driver, *c*, reached high values during the initial years after its introduction (within 300 weeks post-invasion) favoring the invading *D. gazella*. This result is highlighted by the decrease of the fraction of dwellers, $f_{dwell}$, which reached a minimum that also marked the highest dominance of tunnelers. Although very high temperatures and dry conditions negatively affect *D. gazella* individual growth rates, the invader managed to thrive in such conditions, which may be the reason why it established as a non-native species even in arid ecosystems within Texas and Australia^55^. Although temperature variation has been flagged in Texas and Australia as an influential factor in favoring the establishment of *D. gazella*^56,57^, it has remained unclear until now how temperature and relative humidity influenced long-term shifts in native dung beetle community structure in these locations.

The subsequent decreasing trend for the composite driver, *c*, favored instead the dweller functional group. Our data, however, are not conclusive enough to identify the origin of these climatic changes, although it is possible that they are associated with El-Niño Southern Oscillation, typical in this region^1^.

### Alternative community structures/states

Under climatic conditions with intermediate temperatures and relative humidity, our results show the possibility for either dominance by dwellers or the tunneler *D. gazella*, i.e. bistability, with the invader representing the vast majority of tunnelers. The presence of alternative stable states in this dung beetle community leads to uncertainty about its expected structure for a given range of environmental conditions. For values of the composite driver $c\in [0.4,1.6]$, the community could be either overwhelmingly dominated by the dweller or the invader species; for this same range, native tunnelers consistently represented less than $20\%$ of the pre-invasion community. In this bistable regime, the dominance of either group is possible, and our results suggest that even small fluctuations in temperature or relative humidity could shift the relative abundance one way or the other. Note, however, that the invader seemed to exert a stronger dominance over the community than the dweller, as the latter represented when dominating a smaller proportion of the population than when the former dominated ($f_{dwell}$ was 0.55 and 0.71 for the wells indicating dweller-dominated states, whereas the wells indicating invader-dominated states led to a proportion of invader $1-f_{dwell}$ ~ 0.82).

A plausible explanation for bistability in this climate range is that intermediate temperatures and relatively humid conditions favor both groups, creating circumstances in which either could competitively dominate. Coexistence is reinforced by the fact that the native beetles can explore outside grasslands and utilize dung from animals other than cattle, whereas the invader is specialized to open areas and use of livestock dung^40,58^, which contributes to niche separation and reduces competition across groups. Climatic changes that are non-extreme, however, could tilt dominance towards one versus the other group, at least when those changes occur while the community is still in the transient years after invasion (as in our focal example).

In the presence of the invader, large values of the climatic driver (high temperatures and/or low relative humidity) lead to the overwhelming single dominance by tunnelers because, on one hand, dung piles are hotter and drier, which is detrimental for dwellers as they consume food at the surface of the dung pile; on the other hand, the plasticity in climate tolerance of the tunneler *D. gazella* allows it to thrive. In other words, such conditions enable the growth of one group and hinder the growth of the other, preventing the existence of a second well in the potential. The calculation of potentials thus offers more focused information than observing only how annual abundance (e.g. Fig. S1 and Fig. 1a) and climate variables (Fig. S2) change with time.

The existence of alternative stable states was unaltered by either relaxing or tightening our (conservative) choices for smoothing intensity or the threshold above which we considered weekly data as representative. The latter is especially important, since reduced beetle activity (and therefore fewer observations) occurred mostly in colder weeks; our results show that the reported alternative stable states do not result from removing those weeks (which, in any case, affects only the lowest *c* values). The threshold is, nonetheless, necessary because weeks with low activity/observations may lead to the mischaracterization of the community based on only a few individuals, consequences exacerbated by the fact that we focus on the structure of the community (i.e. on proportions, which are very susceptible to low sampling).

### Irreversibility of the transition

The invasive *D. gazella* survives in a wider range of environments than the native beetles, whereas dwellers can thrive in drier and colder environments where the growth of tunneler populations (including *D. gazella*) is more limited^34,39,43,55^. The phase diagram, however, shows that low values of the composite climatic driver *c* were not enough for dwellers to recuperate dominance over tunnelers when these climate conditions prevailed. Thus, the resurgence of the native dweller beetle community in the years after invasion is not necessarily a sign that the community can be fully restored after the removal of *D. gazella*, as its success depends on climatic conditions.

The invasion resulted in the restructuring of the dung beetle community, with a stronger impact of the invader on tunnelers than on dwellers as it reduced the diversity of resident tunnelers and almost drove them to extinction (see above). This increased impact on beetles with similar feeding behavior may be explained by the the functional closeness of *D. gazella* and the native tunnelers^59^, all part of the same taxonomic group. Moreover, the presence of bistability and associated hysteresis further reinforces the possible irreversibility of the transition to single-species dominance by *D. gazella* that occurs for higher *c*. The discontinuity and bistability of the order parameter in the phase diagram, resembling the brown curve (i.e. hypothesized curve 3) in Fig. 1b, means that the community will respond to climatic changes that increase *c* differently from the way it will respond to changes that decrease the climatic driver. This path dependence may eventually hinder an eventual single dominance by dwellers (for which low *c* values are needed, as noted above).

### Changes in resilience associated with the transition

The probability for the dweller-dominated state to withstand a given climate fluctuation, and/or the ranges of the composite driver *c* that would increase this probability, are represented by the area of the associated wells and how it depends on *c*. Our results suggest that, in the presence of the invader, the dweller-dominated state loses stability as the driver *c* increases, in favor of the tunneler-dominated state. This interpretation of the area of the wells in turn enables a quantitative understanding of the resilience of a particular desirable (e.g. dweller-dominated) state and, therefore, of the community structure. According to this, the resilience of the dweller-dominated state decreased as the composite driver, *c*, increased whereas that of the invader-dominated state increased. In contrast, before invasion the stability of the dweller-dominated state remained barely affected by the climatic driver, i.e. the community structure remained resilient against environmental change. Bistability can be expected for any range of the driver for which the areas of both dweller-dominated and tunneler-dominated wells are different from zero, which for our system occurs for intermediate values of *c* after invasion.

### Limitations

Characterizing ecological transitions is very data-intensive. Despite compiling more than 1300 weekly observations spanning 26 years, we needed to use a high smoothing factor to eliminate low-sampling noise that occurred especially for extreme values of the climatic driver *c* and before invasion. Thus, although the bistability we observed is robust from a biological and a technical (e.g. still observed for different smoothing and threshold values) viewpoint, more data would have allowed us to include a wider range for *c* in our analysis and thus investigate whether lower *c* lead to the recovery of single dominance by dwellers even in the presence of the invader.

A longer time period of data collection would also have allowed us to be more certain regarding the behavior of the system before invasion. Our data indicate that, before the invasion, the dweller functional group dominated under all climatic conditions experienced in this time frame. The potentials show that changes in the value of the composite driver *c* did not benefit resident tunnelers (or were so detrimental to dwellers) as to shift the community structure. Also, bistability was not observed before invasion despite the apparent lack of trend shown by the driver, *c*. This suggests that the invasion changed how the community responded to climatic changes: before invasion, the dominance of dwellers would be robust against changes in temperature and relative humidity within ranges for which the community post-invasion showed bistability. The observations before invasion (200 weekly observations), although a rare feat in invasion studies, were still sparse in the context of regime shift analysis and therefore our conclusions regarding the period pre-invasion are to be taken with caution.

Another aspect that calls for caution is the potential existence of other factors that may participate as a driver of the transition. We focused on the combination of temperature and relative humidity because both are important factors influencing beetle survival, and were identified in^1^ as correlating with changes in the number of dwellers and tunnelers. The robustness of our post-invasion results further justifies our choice of driver. However, other factors may also correlate with changes in the number of beetles that we could not include here due to lack of information, such as natural predators targeting the invader after it started dominating the community. Moreover, although not the scope of this study, the co-occurrence of climatic changes and the invasion prevented us from discerning the effects of these two factors separately.

## Conclusions

Our results constitute evidence for the effects of climatic change on the establishment of new exotic terrestrial arthropods. Our framework provides information about the climatic conditions that will favor dominance by either dung beetle functional group, and hence the conditions are more optimal for each group. Recent evidence shows that the area of study (and, more broadly, the Brazilian Cerrado), is becoming warmer and drier^60^, conditions that our study indicates should favor the dominance of *D. gazella*.

Although we applied our methodology to a specific case study, it should be similarly useful in other systems involving biological invasions and climatic change. Given the increased concern on the effects of climate change on ecosystems and the widespread introduction of non-native species, our study provides a framework to assess how these dual forces of global change can interact to cause regime shifts. Climate change may either prevent or favor recently introduced species, the latter disrupting the structure of local communities and leading to augmentative shifts in the stability of ecological systems, which in turn modifies relationships of dominance and may cause loss of biodiversity. In such cases, like in our study, the biological invasion is regulated by climate factors, and therefore it is not possible to propose a management plan to reverse the associated shifts. However, by identifying the key elements involved in these shifts, our framework helps pinpoint and prioritize vulnerable species or groups in the focal system^61^. This information, together with the identification of the order and control parameters associated with the regime shift, can help managers and stakeholders devise strategies designed not to stop the (climate-driven) transition, but rather to ameliorate its potentially disruptive consequences. For example, ecosystem engineering may help eliminate bistability totally or partially^48^, thus reducing uncertainty and increasing the effectiveness of management for invasive species impacts. Thus, our methodology provides relevant information for regional land management, increasing the capacity of local human communities to anticipate regime shifts and reduce the likelihood of associated disruptive consequences^7,28–30^.

## Supplementary Information

Supplementary Information.

## Data Availability

No new data were generated or analyzed in this study. The code that support the findings of this study is available from the corresponding author (J.A.B.), and the data originally collected for^1^ are available from W.M.F. and C.A.H.F., upon request.

## References

[1] Mesquita Filho W, Flechtmann CAH, Godoy WAC, Bjornstad ON (2018). The impact of the introduced *Digitonthophagus gazella* on a native dung beetle community in: Brazil during 26 years. Biol. Invas..

[2] Lewontin RC (1969). The meaning of stability. Brookhaven Symp. Biol..

[3] Holling CS (1993). Resilience and stability of ecological systems. Annu. Rev. Ecol. Syst..

[4] Turner MG, Calder WJ, Cumming GS, Hughes TP, Jentsch A, LaDeau SL, Lenton TM, Shuman BN, Turetsky MR, Ratajczak Z, Williams JW, Williams AP, Carpenter SR (2020). Climate change, ecosystems and abrupt change: Science priorities. Trans. R. Soc..

[5] Albrich K, Rammer W, Turner MG, Ratajczak Z, Braziunas KH, Hansen WD, Seidl R (2020). Simulating forest resilience: A review. Glob. Ecol. Geogr..

[6] Bertani I, Primicerio R, Rossetti G (2016). Extreme climatic event triggers a lake regime shift that propagates across multiple trophic levels. Ecosystems.

[7] Gaertner M, Biggs R, Te Beest M, Hui C, Molofsky J, Richardson DM (2014). Invasive plants as drivers of regime shifts: Identifying high-priority invaders that alter feedback relationships. Divers. Distrib..

[8] Hansen BB, Isaksen K, Benestad RE, Kohler J, Pedersen AO, Loe LE, Coulson SJ, Larsen JO, Varpe Ø (2014). Warmer and wetter winters: Characteristics and implications of an extreme weather event in the High Arctic. Environ. Res. Lett..

[9] Vindstad OPL, Jepsen JU, Ek M, Pepi A, Ims RA (2018). Can novel pest outbreaks drive ecosystem transitions in northern-boreal birch forest?. J. Ecol..

[10] Lin BB, Petersen B (2013). Resilience, regime shifts, and guided transition under climate change: Examining the practical difficulties of managing continually changing systems. Ecol. Soc..

[11] Frelich LE, Reich PB (2009). Will environmental changes reinforce the impact of global warming on the prairie-forest border of central North America?. Front. Ecol. Environ..

[12] Ratajczak Z, Carpenter SR, Ives AR, Kucharik CJ, Ramiadantsoa T, Stegner MA, Williams JW, Zhang J, Turner MG (2018). Abrupt change in ecological systems: Inference and diagnosis. Trends Ecol. Evol..

[13] MacNally R, Nerenberg S, Thomson JR, Lada H, Clarke HR (2014). Do frogs bounce, and if so, by how much? Responses to the ‘Big Wet’ following the ‘Big Dry’ in southeastern Australia. Glob. Ecol. Biogeogr..

[14] Mumby PJ, Bejaran S, Golbuu Y, Steneck RS, Arnold SN, van Woesik R (2013). Empirical relationships among resilience indicators on Micronesian reefs. Coral Reefs.

[15] Garnier A, Hulot FD, Petchey OL (2020). Manipulating the strength of organ ism-environment feedback increases nonlinearity and apparent hysteresis of ecosystem response to environmental change. Ecol. Evol..

[16] Schlax K, Goldenfeld N (2013). Critical Transitions in Ecology.

[17] Schröder A, Persson L, De Roos AM (2005). Direct experimental evidence for alternative stable states: A review. Oikos.

[18] Nascimento, Y. A., Bianchin, I. & Honer, M. R. Instruções para a criação do besouro africano *Onthophagus gazella* em laboratório. Tech. rep. EMBRAPA, p. 33 (1990)

[19] Bianchin I, Honer MR, Gomes A (1992). Controle integrado da mosca-dos-chifres na regia Centro-Oeste. A Hora Vet.

[20] Beynon WA, Wainwright SA, Christie M (2015). The application of an ecosystem services framework to estimate the economic value of dung beetles to the U.K. cattle industry. Ecol. Entomol..

[21] Doube B (2008). Ecosystem services provided by dung beetles in Australia. Basic Appl. Ecol..

[22] Penttilä A, Slade EM, Simojoki A, Riuttaa T, Minkkinen K, Roslin T (2013). Quantifying beetle-mediated effects on gas fluxes from dung pats. PLoS One.

[23] Slade EM, Riutta T, Roslin T, Tuomisto HL (2016). The role of dung beetles in reducing greenhouse gas emissions from cattle farming. Nature.

[24] Iwasa M, Moki Y, Takahashi J (2015). Effects of the activity of coprophagous insects on greenhouse gas emissions from cattle dung pats and changes in amounts of nitrogen, carbon, and energy. Environ. Entomol..

[25] Milotić T (2018). Functionally richer communities improve ecosystem functioning: Dung removal and secondary seed dispersal by dung beetles in the Western Palaearctic. J. Biogeogr..

[26] Halffter G, Matthews EG (1966). The natural history of dung beetles of the subfamily Scarabaeinae (Coleoptera, Scarabaeidae). Folia Entomol. Mex..

[27] Bornemissza GF (1960). Could dung eating insects improve our pasture?. J. Aust. Inst. Agric. Sci..

[28] Pejchar L, Mooney HA (2009). Invasive species, ecosystem services and human well-being. Trends Ecol. Evol..

[29] Kueffer C, Pysek P, Richardson D (2013). Integrative invasion science: Model systems, multi-site studies, focused meta-analysis and invasion syndromes. New Phytol..

[30] Chaffin BC, Garmestani AS, Gunderson LH, Benson MH, Angeler DG, Arnold CA, Cosens B, Craig RK, Ruhl JB, Allen CR (2016). Transformative environmental governance. Annu. Rev. Environ. Resour..

[31] Tarasov S, Dimitrov D (2016). Multigene phylogenetic analysis redefines dung beetles relationships and classification (Coleoptera: Scarabaeidae: Scarabaeinae). BMC Evol. Biol..

[32] Fletchmann CAH, Rodrigues SR, Couto HTZ (1995). Controle biologico da mosca-dos chifres (Haematobia irritans irritans) em Selviria, Mato Grosso do Sul. Acao de insetos fimicolas em massas fecais no campo. Revista Brasileira de Entomologia.

[33] Ratte HT, Hoffmann KH (1984). Environmental physiology and biochemistry of insects. Temperature and Insect Development.

[34] Rodrigues, S. R. & Marchini, L. C. Besouros coprofagos (Coleoptera; Scarabaeidae) coletados em Piracicaba, SP. *Sci. Agric.* (1998).

[35] Floate, K. D., Watson, D. W., Coghlin, P. & Olfert, O. Degree-day models for development of the dung beetles *Onthophagus nuchicornis*, *O. taurus*, and *Digitonthophagus gazella* (Coleoptera: Scarabaeidae), and the likelihood of *O. taurus* establishment in southern Alberta, Canada. *Can Entomol.*, 1–11 (2014).

[36] Krell-Westerwalbesloh S, Krell F-T, Linsenmair E (2004). Diel separation of Afrotropical dung beetle guilds-avoiding competition and neglecting resources (Coleoptera: Scarabaeoidea). J. Nat. Hist..

[37] Greenham PM (1972). The effect of the temperature of cattle dung on the rate of development of the larvae of the Australian bushfly, *Musca vetustissima* Walker (Diptera: Muscidae). J. Anim. Ecol..

[38] Hanski I, Cambefort Y (1991). Dung Beetle Ecology.

[39] Cabrero-Sañudo FJ, Lobo JM (2009). Biogeography of Aphodiinae dung beetles based on the regional composition and distribution patterns of genera. Biogeography.

[40] Genier F, Moretto P (2017). *Digitonthophagus* Balthasar, 1959: Taxonomy, systematics, and morphological phylogeny of the genus revealing an African species complex (Coleoptera: Scarabaeidae: Scarabaeinae). Zootaxa.

[41] Pablo-Cea JD, Velado-Cano MA, Fuentes R, Cruz M, Noriega JA (2017). First report of *Digitonthophagus gazella* (Fabricius, 1787) and new records for *Euoniticellus intermedius* (Reiche, 1849) (Coleoptera: Scarabaeidae Latreille, 10802) in El Salvador. Acta Zool. Mex..

[42] Silveira Neto, S. & Silveira, A. C. “Armadilha luminosa modelo “Luiz de Queiroz”. *O Solo*, pp. 19–21 (1969).

[43] Fletchmann CAH, Rodrigues SR, Couto HTZ (1995). Controle biologico da mosca-dos-chifres (*Haematobia irritans irritans*) em Selviria, Mato Grosso do Sul. 4. Comparação entre métodos de coleta de besouros coprófagos (Scarabaeidae). Revista Brasileira de Entomologia.

[44] Yamamura E (2015). The impact of natural disasters on income inequality: Analysis using panel data during the period 1970 to 2004. Int. Econ. J..

[45] Cleveland WS, Grosse E, Shyu WM, Chambers JM, Hastie TJ (1992). Local regression models. Statistical Models in S.

[46] Shi, J. Q., Wang, B., Will, E. J. & West, R. M. Mixed-effects Gaussian process functional regression models with application to dose–response curve prediction. *Stat. Med.* (2012). 10.1002/sim.4502 22865484

[47] R Core Team. *R: A Language and Environment for Statistical Computing* (R Foundation for Statistical Computing, 2019).

[48] Villa Martin P, Bonachela JA, Levin SA, Muñoz MA (2015). Eluding catastrophic shifts. Proc. Natl. Acad. Sci..

[49] Scheffer M, Carpenter S, Foley JA, Folke C, Walker B (2001). Catastrophic shifts in ecosystems. Nature.

[50] Lenton TM, Held H, Kriegler E, Hall JW, Lucht W, Rahmstorf S, Schellnhuber HJ (2008). Tipping elements in the Earth’s climate system. Proc. Natl. Acad. Sci..

[51] Menck PJ, Heitzig J, Marwan N, Kurths J (2013). How basin stability complements the linear-stability paradigm. Nat. Phys..

[52] Walker, B., Holling, C. S., Carpenter, S. R. & Kinzig, A. Resilience, adaptability and trans formability in social–ecological systems. *Ecol. Soc.* **9**(2) (2004). http://www.ecologyandsociety.org/vol9/iss2/art5/.

[53] Vasilakopoulos P, Marshall CT (2015). Resilience and tipping points of an exploited fish population over six decades. Glob. Change Biol..

[54] Blackburn TM, Pysek P, Bacher S, Carlton JT, Duncan RP, Jarosik V, Wilson JRU, Wilson JRU, Richardson DM (2011). A proposed unified framework for biological invasions. Trends Ecol. Evol..

[55] Noriega JA, Floate KD, Genier F, Reid CAM, Kohlmann B, Horgan FG, Davis ALV, Forgie SA, Aguilar C, Ibarra MG, Vaz-de-Mello F, Ziani S, Lobo JM (2020). Global distribution patterns provide evidence of niche shift by the introduced African dung beetle *Digitonthophagus gazella*. Entomol. Exp. Appl..

[56] Howden HF, Scholtz CH (1986). Changes in a Texas dung beetle community between 1975 and 1985 (Coleoptera: Scarabaeidae, Scarabaeinae). Coleoptera Bull..

[57] Duncan RP (2016). How propagule size and environmental suitability jointly determine establishment success: A test using dung beetle introductions. Biol. Invas..

[58] Tissiani ASO, Vaz-de-Mello FZ, Campelo-Júnior JH (2017). Dung beetles of Brazilian pastures and key to genera identification (Coleoptera: Scarabaeidae). Pesquisa Agropecuaria Brasileia.

[59] Duncan RP, Williams PA (2002). Ecology: Darwin’s naturalization hypothesis challenged. Nature.

[60] Hofmann GS, Cardoso MF, Alves RJV, Weber EJ, Barbosa AA, de Toledo PM, Pontual FB, de Salles Leandro O, Hasenack H, Cordeiro JLP, Aquino FE, de Oliveira LFB (2021). The Brazilian Cerrado is becoming hotter and drier. Glob. Change Biol..

[61] Hulme PE (2017). Climate change and biological invasions: Evidence, expectations, and response options. Biol. Rev..

