# Supplementary material for: Alternative stable ecological states observed after a biological invasion
Source: Sci Rep. 2022 Dec 2;12:20830. doi: 10.1038/s41598-022-24367-3 (PMC9718761; doi:10.1038/s41598-022-24367-3)
Supplement: Supplementary file 1 — Supplementary Information. [file 41598_2022_24367_MOESM1_ESM.pdf]

Alternative stable ecological states observed after a biological invasion

Supplementary Information

Adriano G. Garcia[1] and , Walter Mesquita-Filho[2], Carlos A.H. Flechtmann[3], Julie L. Lockwood[1], and Juan A. Bonachela[1,*]

[1]Department of Ecology, Evolution, and Natural Resources, Rutgers University, New Brunswick 08901, U.S.A.
[2]Departamento de Entomologia e Acarologia, Escola Superior de Agricultura Luiz de Queiroz, Universidade de São Paulo (USP), Piracicaba, SP, CEP 13418-900, Brazil
[3]Departamento de Fitossanidade, Engenharia Rural e Solos, Faculdade de Engenharia, Universidade Estadual Paulista (UNESP), Ilha Solteira, SP, CEP 15385-00, Brazil
[*]To whom correspondence should be addressed: juan.bonachela@rutgers.edu

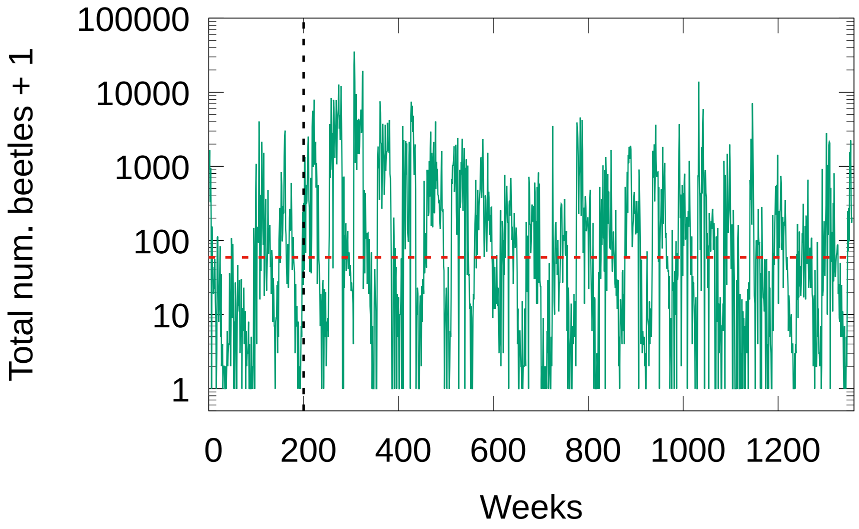

Figure S1: Total number of beetles collected over weeks [1]. The black dashed line indicates the moment of invasion of *D. gazella* and the red dashed line indicates the median of all data. Note that the vertical axis is in logarithmic scale (which required adding 1 to the variable to avoid zeros).

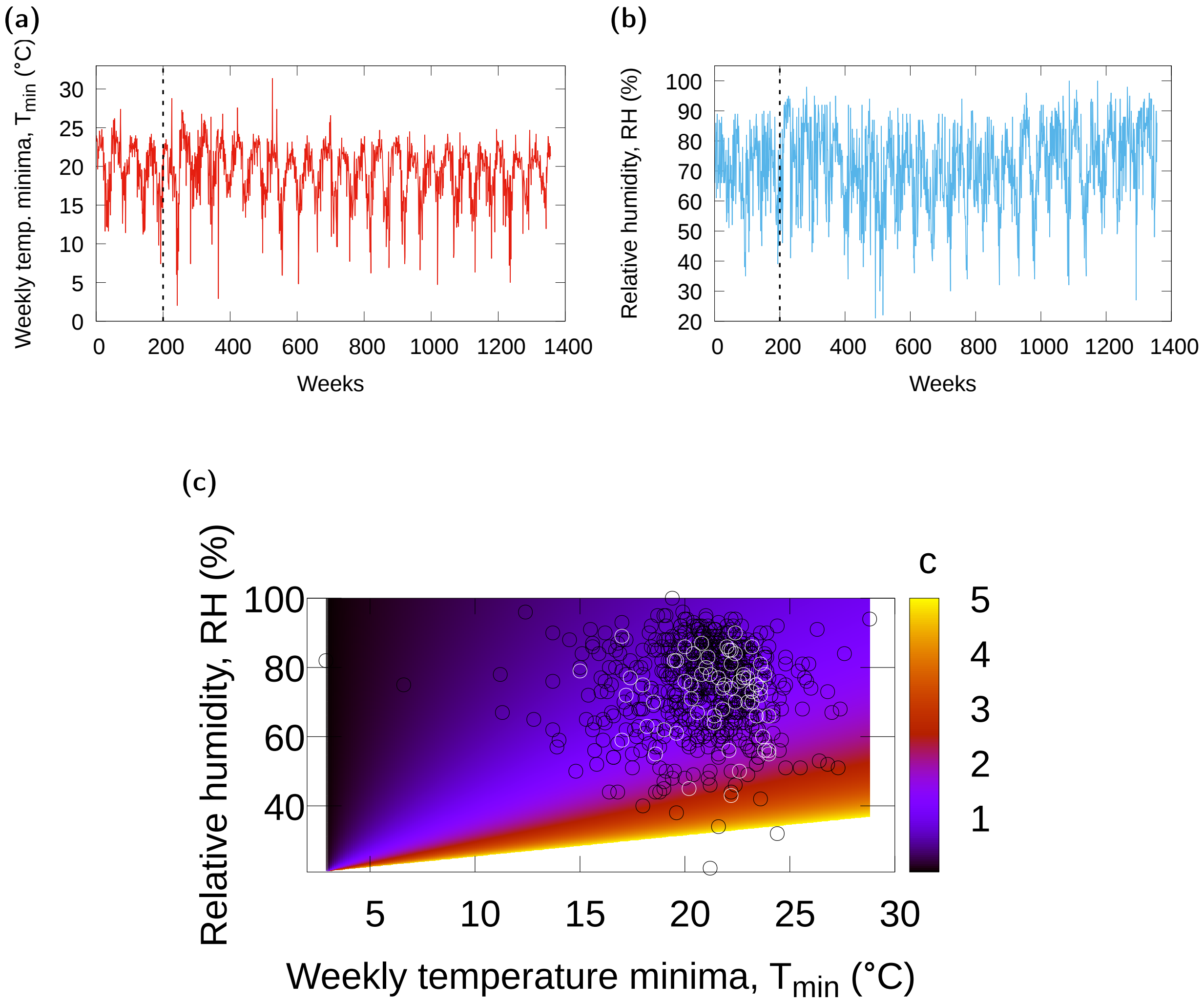

Figure S2: Relationship between the value for the climatologic variables measured in the study site and resulting c. $T_{min} = [2.9°C, 28.8°C]$ and $RH = [45\%, 96\%]$ before the invasion; $T_{min} = [6.6°C, 27.6°C]$ and $RH = [22\%, 100\%]$ after the invasion. Left: temperature. Right: Relative humidity. Center: Resulting value of c for each pair (T_{min},RH), with white circles representing the period before invasion and black circles the period after invasion.

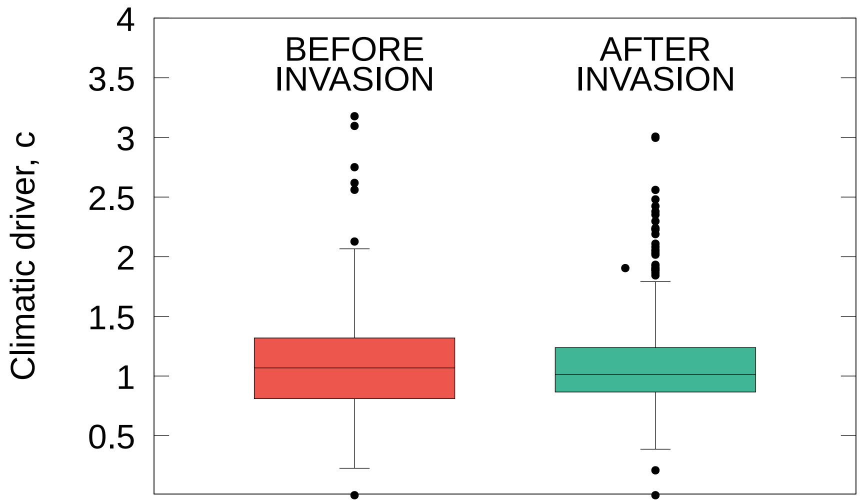

Figure S3: Boxplot for the climatic driver, c, before and after invasion. The top and bottom of each box represent the 25th and 75th percentile, the horizontal bar within the box represents the 50th percentile (i.e. median), and whiskers cover 1.5 times the interquartile range. The dots represent outliers (four points, with $c = 4.4$, $c = 5.5$, $c = 5.9$, and $c = 55.8$ have been removed to ensure readability of the plot).

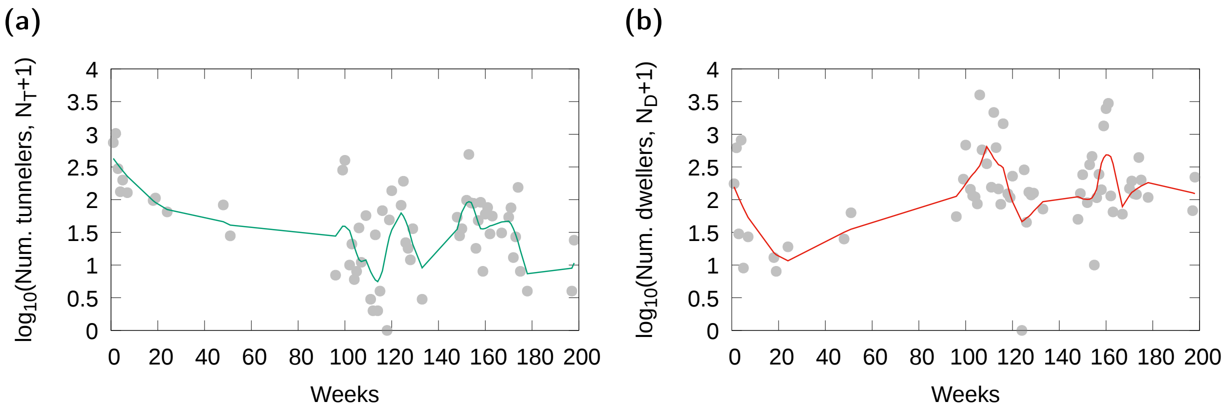

Figure S4: Log-transformed number of individuals for all tunnelers (left) and dwellers (right) as a function of time before the invasion. The curve through the points was obtained using a nonparametric local regression smoother (LOESS) with smoothing intensity 0.25.

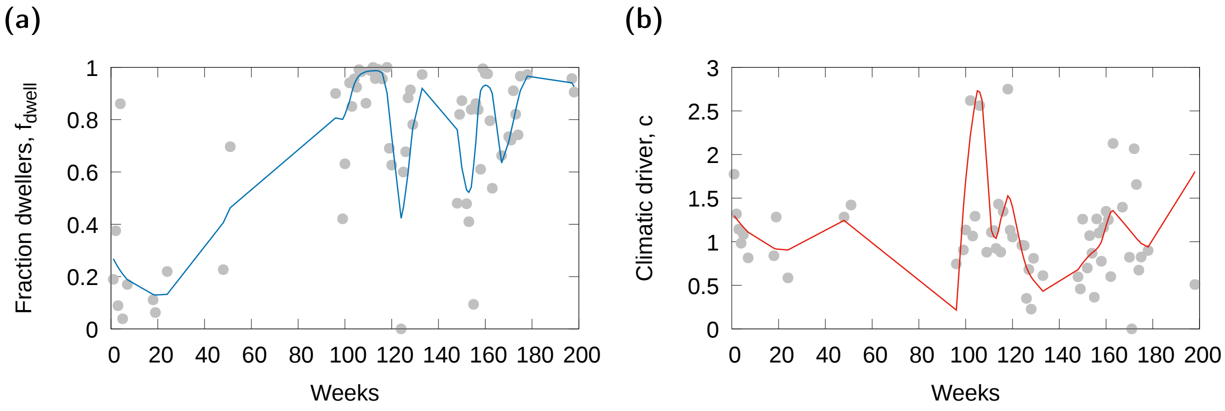

Figure S5: Change with time (weeks before invasion) of a) the fraction of dwellers, f_{dwell}, b) climate observable, c.

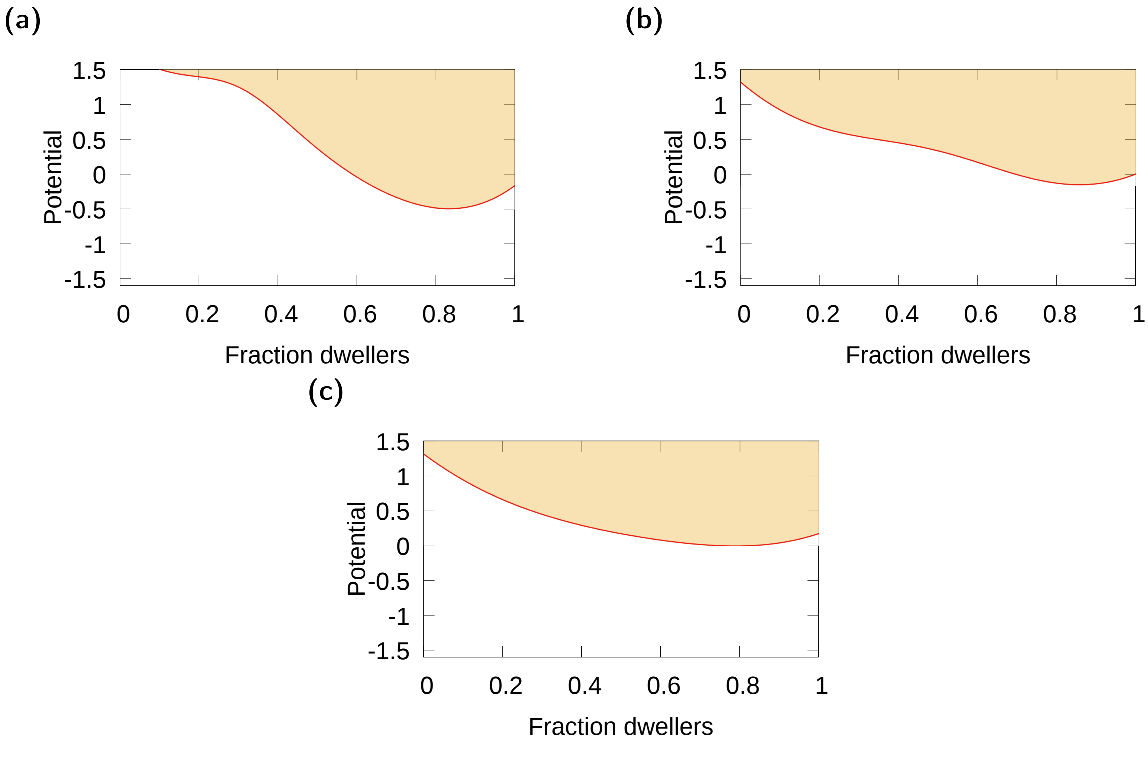

Figure S6: Potentials calculated for different values of c before invasion (i.e. system dwellers - tunnelers), calculated as explained above. a) $T_{min} = [17 - 21.1]$, $RH = [59 - 87]$; b) $T_{min} = [18.6 - 23.6]$, $RH = [55 - 90]$; c) $T_{min} = [21.4 - 23.8]$, $RH = [64 - 78]$. The complete information on the bins can be verified in Table S1.

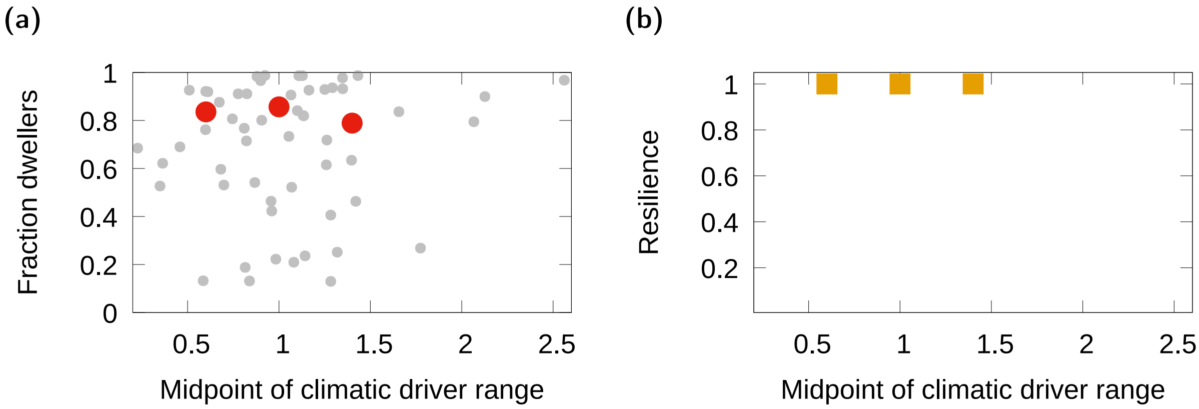

Figure S7: Left: Phase diagram, i.e. relationship between f_{dwell} and c before invasion; red points represent the location of the minima in the associated potentials (see Fig. 4). Right: Area of the wells represented in the different potentials; squares represent the dweller-dominated well and circles the tunneler-dominated well.

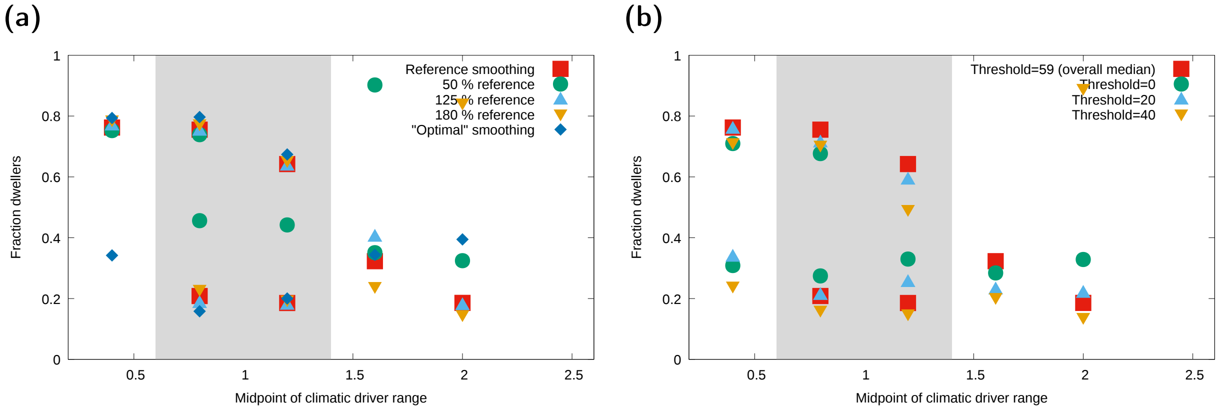

Figure S8: Phase diagram for various choices of smoothing intensity and data-removal thresholds, which did not alter our main conclusions. Left: Smoothing intensities above and below our choice (0.25) show bistability, i.e. the presence of alternative stable states, with extreme values extending the original bistability region (shaded area) also to high values of the climatic driver parameter, c. Right: Removing weeks under a threshold that is higher or lower than our choice (median of all data, $N_{median} = 59$) still leads to bistability, with lower values for the threshold extending the bistability region also to low c values.

Table S1: Information corresponding to each bin used to calculate the probability distributions and associated potentials: number of points, T_{min}, RH and median of beetles (dwellers + tunnelers). Note that extreme bins were merged to the previous/next one (indicated with the "larger-than" and "lower-than" signs), but not used due to the reduced number of points.

Period after invasion					
c	points	T_{min} range ($^{\circ}C$)	T_{min} avg. ($^{\circ}C$)	RH range (%)	RH avg. (%)
< 0.4	3	-	-	-	-
0.4 - 0.8	84	11.2 - 21.6	18.2	65 - 100	86.2
0.8 - 1.2	355	13.7 - 28.8	21.1	56 - 94	79
1.2 - 1.6	125	14.8 - 27.4	22	50 - 77	65
1.6 - 2.0	27	16.4 - 24.6	21.8	44 - 61	54
2.0 - 2.4	12	18.6 - 26.8	22.6	44 - 53	49
> 2.4	8	-	-	-	-
Period before invasion					
c	points	T_{min} range ($^{\circ}C$)	T_{min} avg. ($^{\circ}C$)	RH range (%)	RH avg. (%)
< 0.4	4	-	-	-	-
0.4 - 0.8	11	17 - 21.1	19.4	59 - 87	77
0.8 - 1.2	25	18.6 - 23.6	21.6	55 - 90	77
1.2 - 1.6	12	21.4 - 23.8	22.9	64 - 78	71
> 1.6	10	-	-	-	-
